# The Care of the Feet

**Published:** 1898-07-16

**Authors:** 


### THE CARE OF THE FEET.

The Americans are already experiencing what has troubled every great army from the earliest times in the history of shoe leather, namely, that boots are a great difficulty. By giving a good price and by careful inspection the quality may, no doubt, be kept up to the mark; but the fit is always a trouble, for although it is true enough that, taking men by the thousand, the feet of the vast majority do conform with certain standards as to size and shape, there is always a very considerable minority who require much care in fitting, and a perhaps still larger number who, however well their boots may fit, are always tender footed. This tender footedness is no doubt connected with the growth of micro-organisms; the warmth and moisture of the foot making the soldier's boot an almost ideal incubator, while the organic substances contained in the perspiration provide a constant supply of nutrient medium for the development of bacteria. Much care and labour have been expended in the investigation of the microbes which are connected with sore, and incidentally with perspiring and offensive feet. There is much reason to believe that the organisms engaged are many and various. But in any case there can be no doubt that the hot, steamy foot is much more likely than the firm, but dry foot to become sore, partly from mechanical causes connected with the sodden condition of its protective covering of epidermis, and partly from the fact that in such cases the skin, the stockings, and the boots themselves become so highly charged with micro-organisms that the slightest abrasion quickly becomes a sore.

Acting on this hypothesis many antiseptics have been employed for the purpose of breaking up the microbic link in the chain of events which so often leads up to the production of sore feet. Salt and water, vinegar, and spirits of wine are old domestic remedies. Washing the feet and the boots with perchloride of mercury, bathing them with carbolic lotions, and especially the free use of boracic acid as a dusting powder, both for feet, for stockings, and for boots, have all been found of service. The *Fusspulver* of the German Army, which consists chiefly of salicylic acid and chromic acid, has a double action, hardening the epidermis and probably checking perspiration, as well as operating as a germicide. With the same object tannoform, a compound of formaldehyde with tannin, has lately been used. It is a powder, and has the advantage of being without smell. It may be used pure or mixed with talc as a puff, or as an ointment with vaseline, but for the purpose now in question is best used dry. The secretion diminishes after the first application of the powder, and the macerated parts quickly recover their natural condition. Probably, however, the tannin is unnecessary, and Adler, Frey, and more recently Gerdeck, have advocated the use of simple formalin (an aqueous solution of formic aldehyde). In cases of offensive feet, Frey has the soles and the interdigital spaces washed once or twice a day with a 2 per cent. solution of formalin, and has the insides of the boot, especially the sole, washed out with the same solution and carefully dried afterwards. In a few days the offensive odour disappears. Gerdeck, however, uses the strong undiluted formalin, and he says that when this has been applied to the feet a few times they do not sweat again for two or three weeks, even though woollen stockings be worn. The undiluted formalin is to be pencilled over the feet three or four times, at intervals of about six hours, and a few drops of the fluid are to be dropped into the boots to disinfect them. It is claimed that, used in this way, it not only acts as a preventative of sore feet, but that even when blistering has occurred it hardens the softened tissues and makes walking painless. Although we cannot speak of this matter as yet from personal experience, there is much in the action of formalin in many branches of surgery to support the claims here put forward. That "sweaty feet" have much to do with the general health no one who has observed these cases can deny. At the same time there is no doubt that the condition is in many cases coincident with the best of health, and that in other cases after all constitutional measures have had fair play the offensive condition remains, notwithstanding the improvement in the health at large. It becomes a local affair, requiring some such local treatment as we have indicated, and it should be noted that although in civil life moist, offensive, and tender feet are not productive of the disastrous consequences that may attend their prevalence in an army, they still are the cause of much physical distress, much mental misery, and much incapacity for work, and that, unattractive though the subject may seem, the medical man who can relieve the condition will not only do much good but will gain much credit.

